# XPF expression and its relationship with the risk and prognosis of colorectal cancer

**DOI:** 10.1186/s12935-020-01710-0

**Published:** 2021-01-06

**Authors:** Huixin Hu, Jingjing Jing, Xiaodong Lu, Yuan Yuan, Chengzhong Xing

**Affiliations:** 1 Tumor Etiology and Screening Department of Cancer Institute and General Surgery, the First Hospital of China Medical University, 155# North Nanjing Street, Heping District, Shenyang City, Liaoning Province 110001 China; 2 Liaoning Provincial Education Department, Key Laboratory of Cancer Etiology and Prevention (China Medical University), Shenyang, 110001 China; 3 Key Laboratory of Gastrointestinal Cancer Etiology and Screening, Shenyang, 110001 Liaoning Province China

**Keywords:** Colorectal cancer, XPF, Expression, Risk, Prognosis

## Abstract

**Background:**

XPF (xeroderma pigmentosum complementation group F) is a key factor contributing to DNA damage excision of nucleotide excision repair pathway. The relationship between XPF expression and the risk and prognosis of colorectal cancer (CRC) is unclear.

**Methods:**

In this experiment, a total of 824 cases of colorectal tissue were collected. XPF protein expression was detected by immunohistochemical staining. We conducted a Mann–Whitney U test in order to explore the differential expression of XPF between CRC and non-cancer controls, and the correlation between XPF expression and CRC clinicopathological parameters. Univariate and multivariate Cox regression analyses were conducted to investigate the relationship between XPF expression and CRC prognosis. The Java based software GSEA as well as STRING, David, GO, KEGG were used to explore the function and regulation network of XPF.

**Results:**

The results demonstrated that the XPF expression in CRC was significantly up-regulated compared with non-tumor controls (P < 0.001) and adenoma tissue (P < 0.001). XPF protein was increased in the dynamic sequence of anal diseases to adenoma tissue to CRC. Expression of XPF was related to tumor location (P = 0.005) and tumor growth pattern (P = 0.009). The results of prognosis analysis suggested that in patients with stage T1-T2, XPF low expression may be significantly associated with better overall survival (HR = 7.978, 95% CI 1.208–52.673, P = 0.031). XPF and its interacting genes played a vital role in different processes of nucleotide excision repair pathway. XPF expression was related with Ubiquitin like protein specific protease activity.

**Conclusions:**

XPF might be a promising biomarker for CRC risk, and also showed potential as a prognostic predictor in CRC patients.

## Background

DNA damage caused by endogenous or exogenous genetic toxicants can contribute to genomic instability and directly lead to a variety of cancers. Cells have evolved a series of DNA repair pathways to avoid the deleterious result [1]. Nucleotide excision repair (NER) can identify many types of underlying damage and cut damaged DNA strands at precise distances on both sides of the lesion, as well as base-damaged oligonucleotide fragments [2]. NER pathway has four main steps which are damage identification, damage partitioning and unwinding, damage incision and new strand synthesis [3, 4]. XPF(xeroderma pigmentosum complementation group F), locating on chromosome 16p13.12, has 11 exons with a span of 28.2 kb [4]. The heterodimer of XPF-ERCC1 is involved with the 5′ incision step of the NER pathway. The catalytic area located in XPF can determine the activity of NER [5]. It is an essential human gene in the NER pathway responsible for the removal of UV-C photoproducts and large volume adducts from DNA [6, 7]. Cells or animals that lack XPF cannot perform the NER pathway [4].

Given its important function in the NER pathway, XPF may be involved in diseases associated with imbalance between DNA damage and repair. A number of researches have focused on its role in different cancers. XPF has been reported in the literature that its expression in renal cell carcinoma is significantly higher compared with bladder cancer and testicular cancer, and is related to the clinical features and chemotherapy sensitivity [6]. XPF expression is increased in gastric cancer (GC) tissue, and the prognosis of patients with high expression of XPF is poor [7]. XPF is also highly expressed in oral cancer tissue, while its high expression indicates a low survival rate [8]. Colorectal cancer (CRC) is a malignant tumor which is the third cause of cancer death in China [9]. There have been some previous studies focusing on the relationship between XPF polymorphism and the risk of CRC. The results showed that there was an association between XPF polymorphisms and the risk of CRC [10–12]. So far, although there have been small sample studies investigating the relationship between XPF expression and the risk of CRC [13], the pathological process from colorectal benign diseases to precancerous lesions to cancer has not been studied, and large sample size studies on the relationship between XPF expression and CRC are needed. In the current study, we first studied the expression tendency of XPF in the progression from anal benign disease to adenoma to CRC. Further, we analyzed the association of XPF expression with clinicopathological parameters and survival of CRC patients, thus to investigate the effect of XPF on development, progression, and prognosis of CRC. By performing bioinformatics analyses, we studied the function and regulation network of ERCC4 in CRC.

## Methods

### Patients and tissue specimens

The design of this study was approved by the Human Ethics Committee of China Medical University. Each subject participated in the study provided the written informed consent. The patients undergoing surgery were from the First Hospital of China Medical University between November 2012 and June 2016. We enrolled a total of 824 cases of colorectal tissue for risk study, including 276 cases of CRC and 284 adjacent non-tumor tissue (248 cases of CRC had survival time, 230 pairs had cancer tissue and its matched adjacent tissue), 202 cases of adenoma and 62 cases of anal disease; and 248 cases of CRC tissue with survival time were used for prognosis study.

We collected the tissue of CRC, which were derived from the histological results, and the collection was according to the World Health Organization standards. The TNM staging of CRC was evaluated based on the International Union Against Cancer (UICC)/United Joint Cancer Committee (AJCC) (7th edition in 2010) [6]. There were 3 cases of CRC patients that needed to be excluded: (1) patients with XP disease; (2) patients who received chemotherapy or radiation therapy before surgery; (3) patients with hereditary nonpolyposis colorectal cancer (HNPCC).

Follow-up study was conducted until April 2018. We performed prognostic analysis of 248 patients enrolled (the follow-up time was 12 to 63 months, the average survival time was 48.15 months, and there was no death). We excluded 14 patients who lacked visits in the OS analysis. Patients with the habit of smoking at least one cigarette a day for at least 1 year were considered to have a history of smoking. In the meantime, the study defined the drinking history as an average daily intake of at least 50 grams of alcohol for at least 1 year. Clinical characteristics of cancer patients included gender, age, whether smoking or drinking, tumor location, TNM stage, invasive extent, lymph infiltrative, distant metastasis, tumor deposit, perineural invasion, vessel carcinoma embolus, growth pattern, differentiation degree, maximum diameter and family history.

### Immunohistochemistry

The tissue was fixed in formalin and embedded in paraffin, then cut into 4 μm thick sections, and the sections were mounted on glass slides [14]. Antigen retrieval was performed after routine dewaxing. The tissue sections were washed with phosphate buffered saline (PBS, pH 7.4). Then the sections were blocked with 10% normal goat serum for 10 min. The expression of XPF protein was detected with mouse anti-XPF monoclonal antibody (ab-85,140, 1: 200 dilution; Abcam, Cambridge, UK), and the primary antibody was used to incubate at room temperature for 1 hour. We spined off the primary antibody on the slice, and then used a biotinylated secondary antibody (goat anti-rabbit antibody, Fujian Maixin) to incubate the tissue for 10 min. The tissue was rinsed with PBS for 10 min. After that, we used streptavidin Biotin-biotin peroxide at a temperature of 24–27 °C for incubating the tissue for 10 min, and stained with DAB (DAB-0031, Maixin City, Fujian Province, China) on a glass slide. When the tissue stain become brown (about 30 s), we rinsed the DAB with PBS. Finally, the slides were dehydrated, the tissue was fixed with resin and the coverslips were covered to observe the staining.

### Evaluation of immunohistochemistry

Two experienced pathologists scored XPF’s expression in different tissue independently, and this process followed the double-blind principle. The pathologists scored the staining intensity and staining area of XPF respectively. If there are differences in the scores of the pathologists, two pathologists will discuss and summarize the final scores. Semi-quantitative scoring criteria were used to assess the expression of XPF. Scoring standard: (1) staining intensity was classified into four levels, including 0 (no staining), 1 (light brown), 2 (brown staining), and 3 (heavy brown staining); (2) percentage of stained cells was divided into: 0(0–5); 1(6–25); 2(26–50); 3(51–75); 4(76–100%). We got the final IS (immunoreactivity score) by multiplying staining intensity and percentage of stained scores. Finally, the IS score was classified as: negative (−), score = 0; weak positivity (+), score = 1–4; medium positivity (++), score = 5–8; and strong positivity (+++), score = 9–12.

### Oncomine analysis

Oncomine, a cancer microarray database and web-based data mining platform, aiming to analyze genome-wide expression for cancer types and provide transcriptome data of cancer tissue [15, 16]. We compared the mRNA expression of XPF in normal colon and rectum tissue vs. colon adenocarcinoma by Oncomine. We choosed 1.5 fold change, *P* value = 0.05 and top 10% gene rank as threshold.

### The function and regulation network of XPF by GO and KEGG analysis

STRING is a database designed to collect, score and integrate all public sources of information on protein–protein interactions [17]. Gene ontology (GO) analysis is a major bioinformatics tool that unifies the characterization of genes and gene products through the three components of biological processes, cell composition and molecular function [18]. Kyoto Encyclopedia of Genes and Genomes (KEGG) is a set of databases whose main purpose is to study genetic pathways, and contains information about biological pathways, genomes, chemicals and diseases [19]. The Database for Annotation, Visualization and Integrated Discovery (DAVID; v.6.8; https://david.ncifcrf.gov/home.jsp; accessed on September 16, 2020) was applied to perform the enrichment analyses of GO and KEGG [20]. DAVID is an online portal that provides comprehensive annotation analysis of large gene lists. GO analysis comprises groups of molecular function, cellular function and biological process [21]. We used STRING to explore the genes closely correlated with XPF. XPF and its interacting genes were enriched and analyzed by David for GO and KEGG pathways, respectively. The ggplot2 package in the R platform (Version 3.6.3) was used to show the obtained results. Gene Set Enrichment Analysis (GSEA) is an analysis method for whole-genome expression profiling chip data, which compares genes with predefined gene sets [22]. By analyzing the gene expression profile data, we can understand the expression status of XPF in a specific functional gene set, and whether there is some statistical significance in this expression status. We searched the expression of XPF in normal and cancerous colorectal tissue in the Oncomine database [15].

### Statistical analysis

All the statistical analyses were conducted using SPSS 20.0 software (IL, Chicago). The difference of XPF expression between CRC and adjacent non-tumor tissue was compared by non-parametric tests. We performed Mann–Whitney U test of nonparametric test to evaluate the relationship between XPF expression and clinicopathological parameters of CRC. Survival analysis was performed by Kaplan–Meier method. When we compared the differences between subgroups, and the log-rank test was used. The Cox proportional hazard model was used to evaluate the effect of XPF expression on CRC prognosis. P < 0.05 was considered as statistically significant.

## Results

### The baseline characteristics of the subjects

A total of 824 cases were included in this study. The baseline characteristics and clinicopathological parameters of anal disease, anenoma and CRC are listed in Table 1. The information was further used for the analysis of the relationship between XPF expression and risk, clinicopathological parameters and prognosis of CRC.

**Table 1:** Basic information of the participants

Characteristics	Anal disease	Adenoma	CRC
Gender^a^			
Male	19	24	143
Female	42	26	105
Age(years)^a^			
> 60	22	24	129
≤ 60	38	26	119
Smoking			
Yes			59
No			189
Drinking			
Yes			40
No			208
Tumor location			
Colon			82
Rectum			166
TNM stage^a^			
I			6
II			89
III			132
IV			18
Invasive extent^a^			
T1-2			44
T3-4			155
Lymph node metastasis			
Positive			148
Negative			100
Distant metastasis			
Positive			64
Negative			184
Perineural invasion			
Positive			148
Negative			100
Vessel carcinoma embolus			
Positive			60
Negative			188
Growth patten			
Infiltrative			152
Nested/cloddy			96
Differentiation degree			
Poor/mucinous			80
Well/moderate			168
Family history			
Positive			43
Negative			205

*CRC* colorectal cancer, *MST* median survival time

^a^Incomplete information

### XPF was highly expressed in CRC tissue compared with non-tumor adjacent tissue

**Fig. 1:**
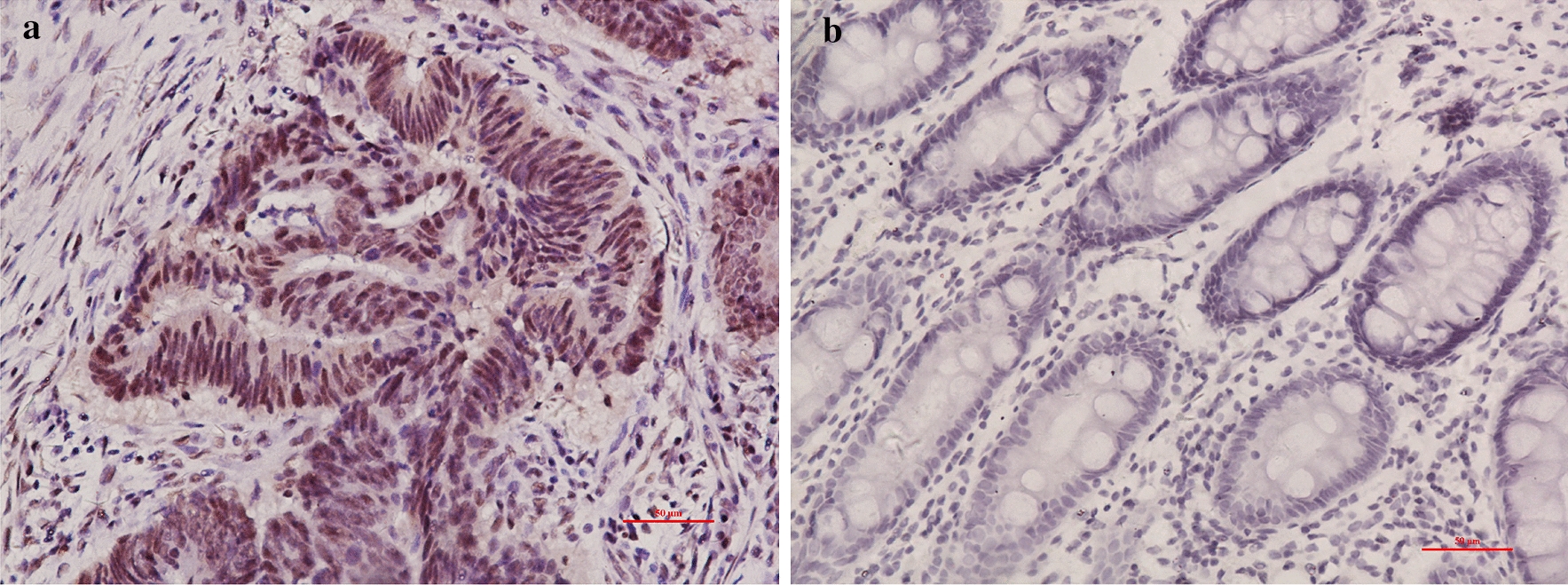
XPF expression in CRC and its matched non-tumor adjacent specimens. XPF expression was detected in the nucleus. **a** Colorectal cancer tissue and **b** adjacent nontumor tissue of CRC. Original magnification, × 200

**Fig. 2:**
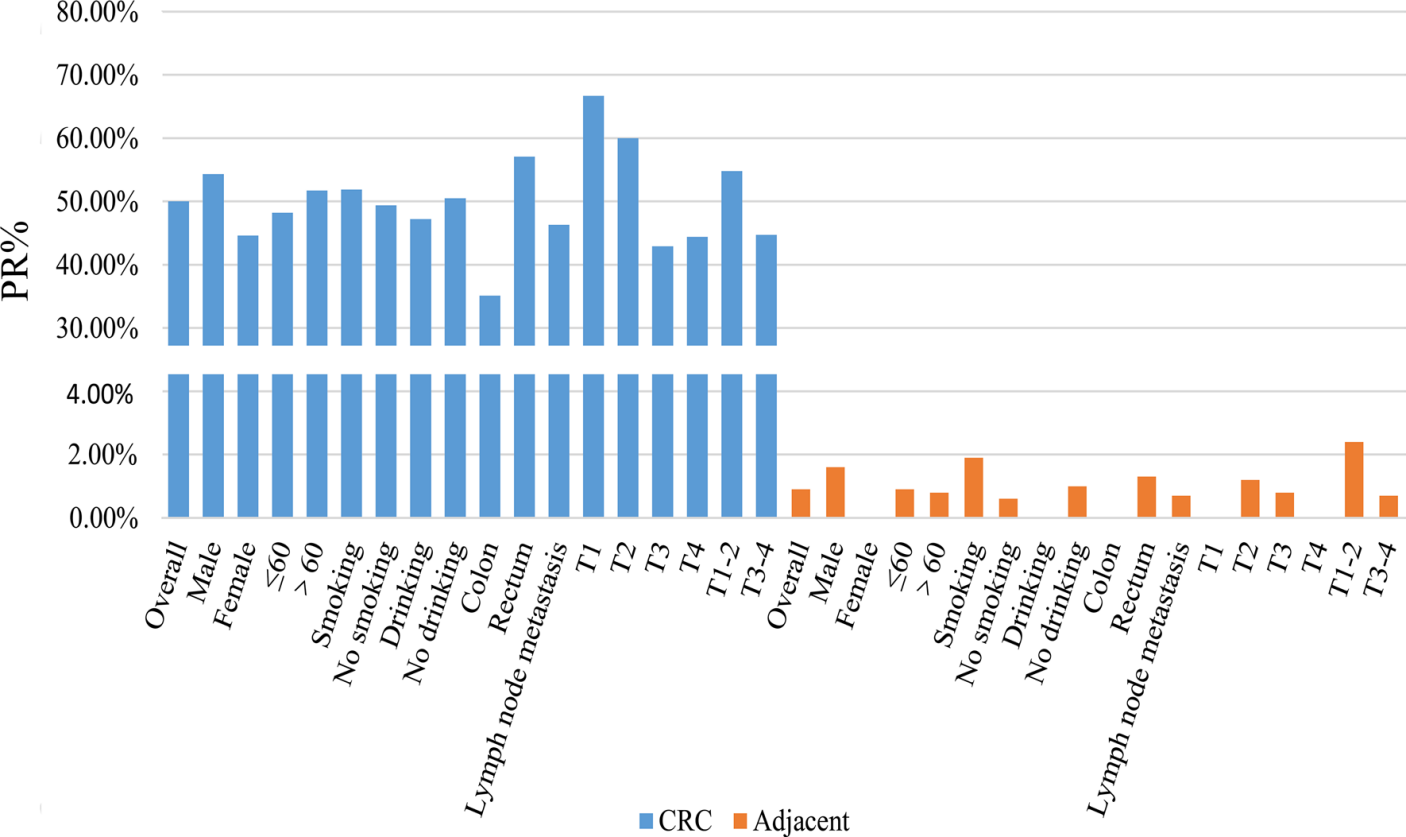
Graphical representation of the results in Table 2, showing the subgroup analysis of XPF expression in CRC and nontumor adjacent tissue

In this study,we enrolled 276 cases of CRC tissue, of which 230 pairs contained cancer and its matched adjacent tissue. The results demonstrated that XPF was highly expressed in CRC tissue compared with adjacent non-tumor tissue (P < 0.001) (Fig. 1). Subgroup analysis showed that XPF expression continued to be significantly up-regulated in cancer tissue in men (P < 0.001), women (P < 0.001), age > 60 (P < 0.001), age ≤ 60 (P < 0.001), colon cancer (P < 0.001), rectal cancer (P < 0.001) and other stratified analysis were meaningful (Table 2 and Fig. 2).

**Table 2:** XPF expression in CRC and nontumor adjacent tissue

Category	Group	Cases	(−)	(+)	(++)	(+++)	PR (%)	P
			n (%)	n (%)	n (%)	n (%)		
Overall	CRC	230	115	94	15	6	50.0%	<*0.001*
	Adjacent	230	228	1	1	0	0.9%	
Male	CRC	129	59	57	9	4	54.3%	<*0.001*
	Adjacent	129	127	1	1	0	1.6%	
Female	CRC	101	56	37	6	2	44.6%	<*0.001*
	Adjacent	101	101	0	0	0	0.0%	
≤60	CRC	112	58	45	7	2	48.2%	<*0.001*
	Adjacent	112	111	1	0	0	0.9%	
>60	CRC	118	57	49	8	4	51.7%	<*0.001*
	Adjacent	118	117	0	1	0	0.8%	
Smoking	CRC	54	26	22	2	4	51.9%	<*0.001*
	Adjacent	54	53	1	0	0	1.9%	
No smoking	CRC	176	89	72	13	2	49.4%	<*0.001*
	Adjacent	176	175	0	1	0	0.6%	
Drinking	CRC	36	19	15	1	1	47.2%	<*0.001*
	Adjacent	36	36	0	0	0	0.0%	
No drinking	CRC	194	96	79	14	5	50.5%	<*0.001*
	Adjacent	194	192	1	1	0	1.0%	
Colon	CRC	74	48	21	3	2	35.1%	<*0.001*
	Adjacent	74	74	0	0	0	0.0%	
Rectum	CRC	156	67	73	12	4	57.1%	<*0.001*
	Adjacent	156	154	1	1	0	1.3%	
Lymph node metastasis	CRC	134	72	51	8	3	46.3%	<*0.001*
	Adjacent	134	133	1	0	0	0.7%	
Without Lymph node metastasis	CRC	96	43	43	7	3	55.2%	<*0.001*
	Adjacent	96	95	0	1	0	1.0%	
Distant metastasis	CRC	57	26	25	3	3	54.4%	<*0.001*
	Adjacent	57	56	1	0	0	1.8%	
No Distant metastasis	CRC	173	89	69	12	3	48.6%	<*0.001*
	Adjacent	173	172	0	1	0	0.6%	
Perineural invasion	CRC	136	72	52	9	3	47.1%	<*0.001*
	Adjacent	136	135	0	1	0	0.7%	
Without Perineural invasion	CRC	94	43	42	6	3	54.3%	<*0.001*
	Adjacent	94	93	1	0	0	1.1%	
Vessel carcinoma embolus	CRC	53	31	19	3	0	41.5%	<*0.001*
	Adjacent	53	53	0	0	0	0.0%	
Without Vessel carcinoma embolus	CRC	177	84	75	12	6	52.5%	<*0.001*
	Adjacent	177	175	1	1	0	1.1%	
Infiltrative	CRC	139	76	54	5	4	45.3%	<*0.001*
	Adjacent	139	138	0	1	0	0.7%	
Nested/cloddy	CRC	91	39	40	10	2	57.1%	<*0.001*
	Adjacent	91	90	1	0	0	1.1%	
Poor/mucinous	CRC	70	41	24	4	1	41.4%	<*0.001*
	Adjacent	70	69	1	0	0	1.4%	
Well/moderate	CRC	160	74	70	11	5	53.8%	<*0.001*
	Adjacent	160	159	0	1	0	0.6%	
Family history	CRC	41	21	14	4	2	48.8%	<*0.001*
	Adjacent	41	40	1	0	0	2.4%	
Without Family history	CRC	189	94	80	11	4	50.3%	<*0.001*
	Adjacent	189	188	0	1	0	0.5%	
T1	CRC	6	2	3	1	0	66.7%	
	Adjacent	6	6	0	0	0	0.0%	*0.022*
T2	CRC	85	34	42	7	2	60.0%	
	Adjacent	85	84	0	1	0	1.2%	<*0.001*
T3	CRC	119	68	43	5	3	42.9%	
	Adjacent	119	118	1	0	0	0.8%	<*0.001*
T4	CRC	18	10	5	2	1	44.4%	
	Adjacent	18	18	0	0	0	0.0%	*0.002*
T1-2	CRC	42	19	18	3	2	54.8%	
	Adjacent	42	41	0	1	0	2.4%	<*0.001*
T3-4	CRC	141	78	51	8	4	44.7%	
	Adjacent	141	140	1	0	0	0.7%	<*0.001*

*PR* positive rate. Negative (−), light positive (+), positive (++), strong positive (+++) staining. Mann–Whitney U- test of nonparametric test to compare the XPF protein expression between CRC and adjacent tissue

The italics values: P < 0.05

**Fig. 3:**
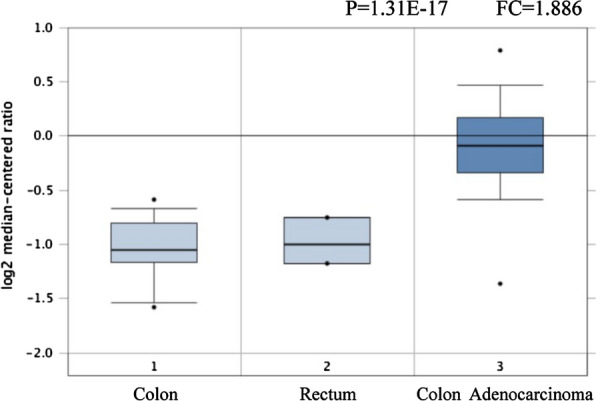
XPF expression in TCGA colorectal cancer cohort from Oncomine database. The cohort are as follows: normal colon cohort, normal rectum cohort, and colon adenocarcinoma cohort. Boxes include 25th–75th percentiles with bars indicating medians; whiskers indicate range of data other than outliers represented by bars. n = 123, P < 0.001

In this study, the mRNA expression of XPF in colorectal cancer and its normal tissue was also analyzed by Oncomine data.The results showed that XPF was highly expressed in CRC compared to normal colon and rectum tissue (P < 0.001). XPF expression had significant relationship with the risk of CRC cancer (Fig. 3).

### XPF was highly expressed in CRC compared with adenoma and anal benign disease

**Fig. 4:**
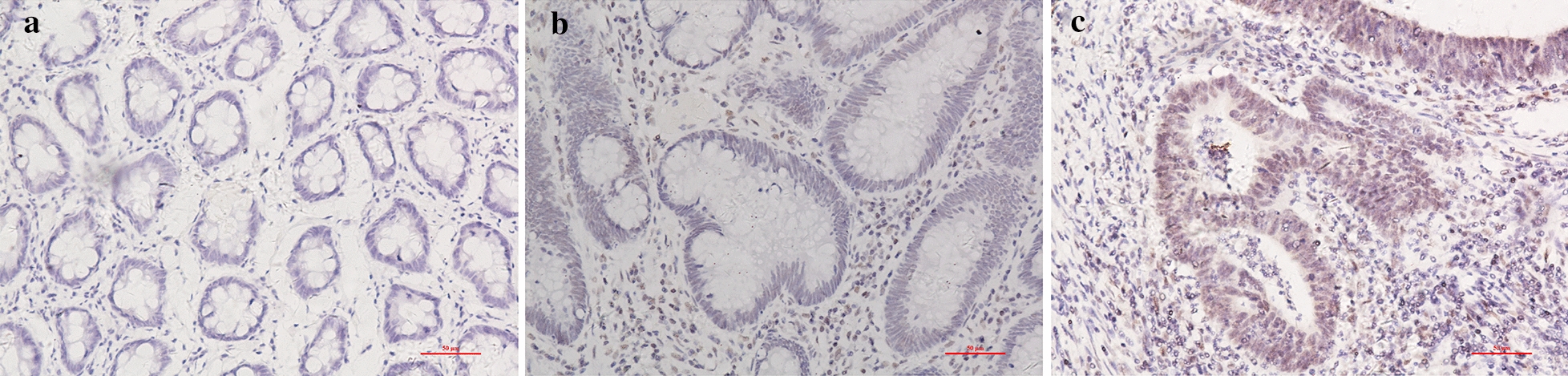
Representative photomicrographs of immunohistochemical staining of XPF in different phase of colorectal diseases. **a** Low nuclear expression of XPF in anal disease. **b** Middle nuclear expression of XPF in adenoma tissue. **c** High nuclear expression in CRC. Showing the dynamic expression in colorectal diseases. Original magnification, × 200

As shown in Fig. 4, XPF expression increased from anal benign disease, adenoma to CRC (P < 0.001). Subgroup analysis demonstrated a similar expression trend of XPF in men (P < 0.001), women (P = 0.001), age ≤ 60 (P = 0.001), age > 60 (P < 0.001) (Table 3).

**Table 3:** XPF expression in CRC, adenoma and anal benign disease tissue

Category	Group	Cases	(−)	(+)	(++)	(+++)	PR (%)	P
			n (%)	n (%)	n (%)	n (%)		
Overall	CRC	276	131	116	17	12	52.5%	<*0.001*
	Adenoma	202	116	67	15	4	42.6%	
	Anal disease	62	55	6	1	0	11.3%	
Male	CRC	143	66	62	9	6	53.8%	<*0.001*
	Adenoma	24	19	4	0	1	20.8%	
	Anal disease	19	17	1	1	0	10.5%	
Female	CRC	105	58	39	6	2	44.8%	*0.001*
	Adenoma	26	16	6	2	2	38.5%	
	Anal disease	42	37	5	0	0	11.9%	
≤60	CRC	119	62	47	7	3	47.9%	*0.001*
	Adenoma	26	18	6	1	1	30.8%	
	Anal disease	38	32	5	1	0	15.8%	
>60	CRC	129	62	54	8	5	51.9%	<*0.001*
	Adenoma	24	17	4	1	2	29.2%	
	Anal disease	22	21	1	0	0	4.5%	
Colon	CRC	82	54	21	3	4	34.1%	0.275
	Adenoma	10	8	2	0	0	20.0%	
Rectum	CRC	166	70	80	12	4	57.8%	0.109
	Adenoma	33	22	6	2	3	33.3%	

*PR* positive rate. Negative (−), light positive (+), positive (++), strong positive (+++) staining. Mann–Whitney U- test of nonparametric test to compare the XPF protein expression between CRC and adjacent tissue

The italics values: P < 0.05

The expression of XPF in CRC was higher than that in benign anal disease (P < 0.001). In the subgroup analysis of the following groups: male (P = 0.001), female (P < 0.001), age ≤ 60 (P < 0.001), age > 60 (P < 0.001), it showed the same result that XPF expression was significantly up-regulated in cancer tissue (Additional file 1: Table S1).

**Fig. 5:**
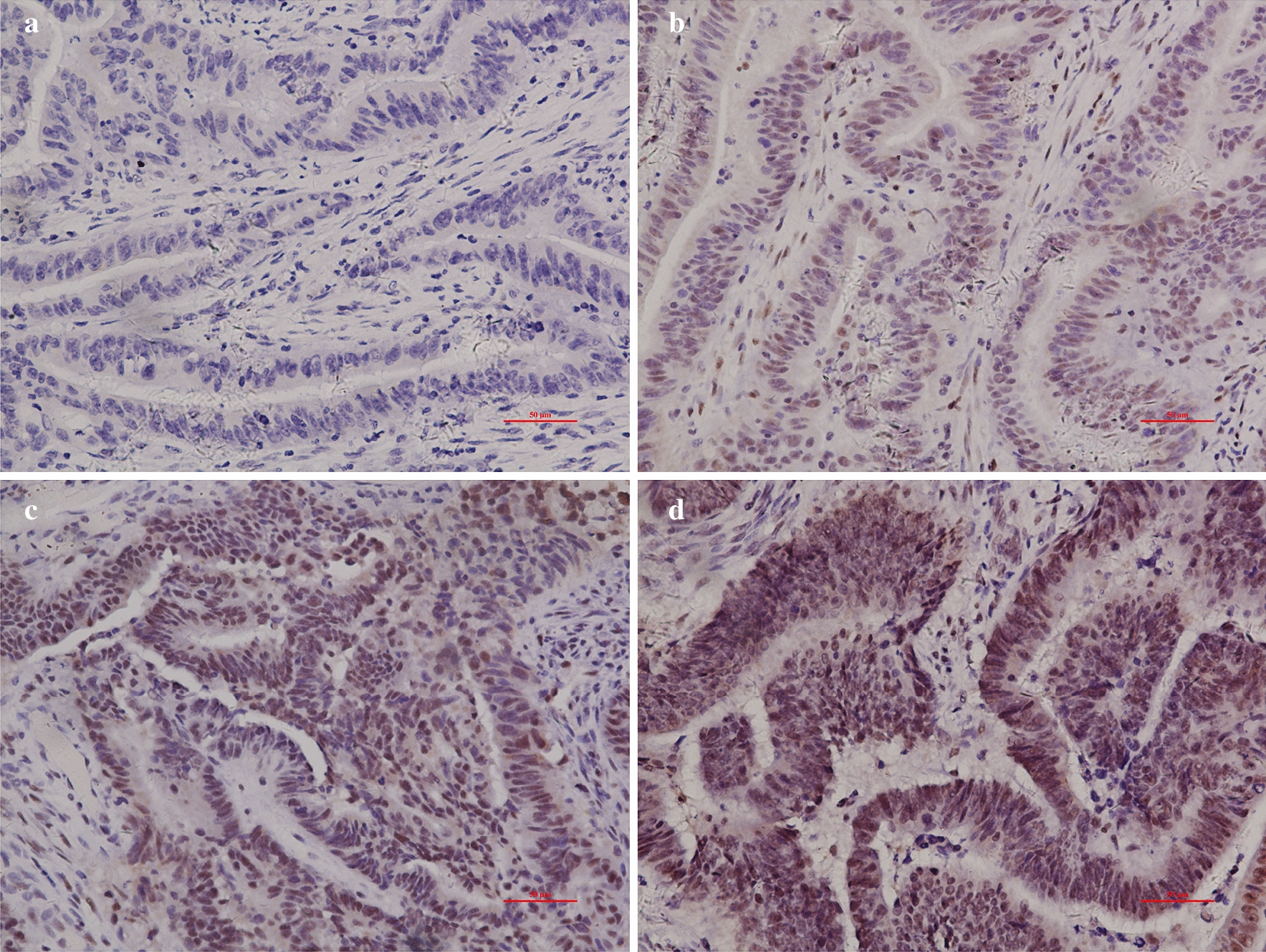
Different levels of XPF expression in CRC tissue. **a** Negative (−),score = 0; **b** weakly positive (+), score = 1–4; **c** moderately positive (++), score = 5–8; **d** strongly positive (+++), score = 9–12

Four different grades of immunoreactivity score (IS) were displayed in Fig. 5, indicating the increasing trend of XPF expression in CRC tissue.

### Relationship between XPF protein expression and clinicopathological parameters in CRC patients

We used the Mann–Whitney U test to study the differences between the XPF groups (Table 4) and stratified the CRC patients based on age, gender, smoking, alcohol consumption, location, TNM stage, invasive extent, lymph node metastasis, distant metastasis, perineural invasion, vessel carcinoma embolus, growth pattern, differentiation degree and family history. The results of stratified analysis showed that XPF protein expression was related to tumor location: XPF expression in rectum cancer patients was higher than that of colon cancer patients (P = 0.005). XPF protein expression was also related to growth patterns: XPF was highly expressed in nested/cloddy CRC compared with infiltrating CRC (P = 0.009). There was a trend that XPF was highly expressed in male than female (P = 0.056). In the meanwhile, XPF was more likely to be expressed in well/moderate CRC than in poor/mucinous CRC (P = 0.083). However, except for the above points, there was no statistical difference for other clinical pathological parameters (P > 0.05).

**Table 4:** Association between XPF expression and clinicopathological parameters in CRC

Variables	Cases	(−)	(+)	(++)	(+++)	PR (%)	P
		n (%)	n (%)	n (%)	n (%)		
Gender							
Male	143	66	62	9	6	53.8%	0.056
Female	105	58	39	6	2	44.8%	
Age (years)							
> 60	119	62	47	7	3	47.9%	0.319
≤ 60	129	62	54	8	5	51.9%	
Smoking							
Yes	59	27	25	2	5	54.2%	0.304
No	189	97	76	13	3	48.7%	
Drinking							
Yes	40	20	17	1	2	50.0%	0.672
No	208	104	84	14	6	50.0%	
Tumor location							
Colon	82	54	21	3	4	34.1%	*0.005*
Rectum	166	70	80	12	4	57.8%	
TNM stage^a^							
I	6	2	3	1	0	66.7%	0.151
II	89	36	44	7	2	59.6%	
III	132	74	48	5	5	43.9%	
IV	18	10	5	2	1	44.4%	
Invasive extent^a^							
T1-2	44	20	19	3	2	54.5%	0.395
T3-4	155	84	57	8	6	45.8%	
Lymph node metastasis							
Positive	148	79	56	8	5	46.6%	0.106
Negative	100	45	45	7	3	55.0%	
Distant metastasis							
Positive	64	30	28	3	3	53.1%	0.341
Negative	184	94	73	12	5	48.9%	
Perineural invasion							
Positive	148	78	57	9	4	47.3%	0.605
Negative	100	46	44	6	4	54.0%	
Vessel carcinoma embolus							
Positive	60	36	20	3	1	40.0%	0.131
Negative	188	88	81	12	7	53.2%	
Growth patten							
Infiltrative	152	85	58	5	4	44.1%	*0.009*
Nested/cloddy	96	39	43	10	4	59.4%	
Differentiation degree							
Poor/mucinous	80	47	27	4	2	41.3%	0.083
Well/moderate	168	77	74	11	6	54.2%	
Family history							
Positive	43	22	15	4	2	48.8%	0.611
Negative	205	102	86	11	6	50.2%	

*PR* positive rate. Negative (−), light positive (+), positive (++), strong positive (+++) staining

The association of XPF expression with TNM stage was analyzed by Kruskal–Wallis H- test of nonparametric test. For other clinicopathological parameters, Mann–Whitney U- test of nonparametric test was used

The italics values: P < 0.05

^a^Incomplete information

### Relationship between XPF expression and CRC prognosis

**Fig. 6:**
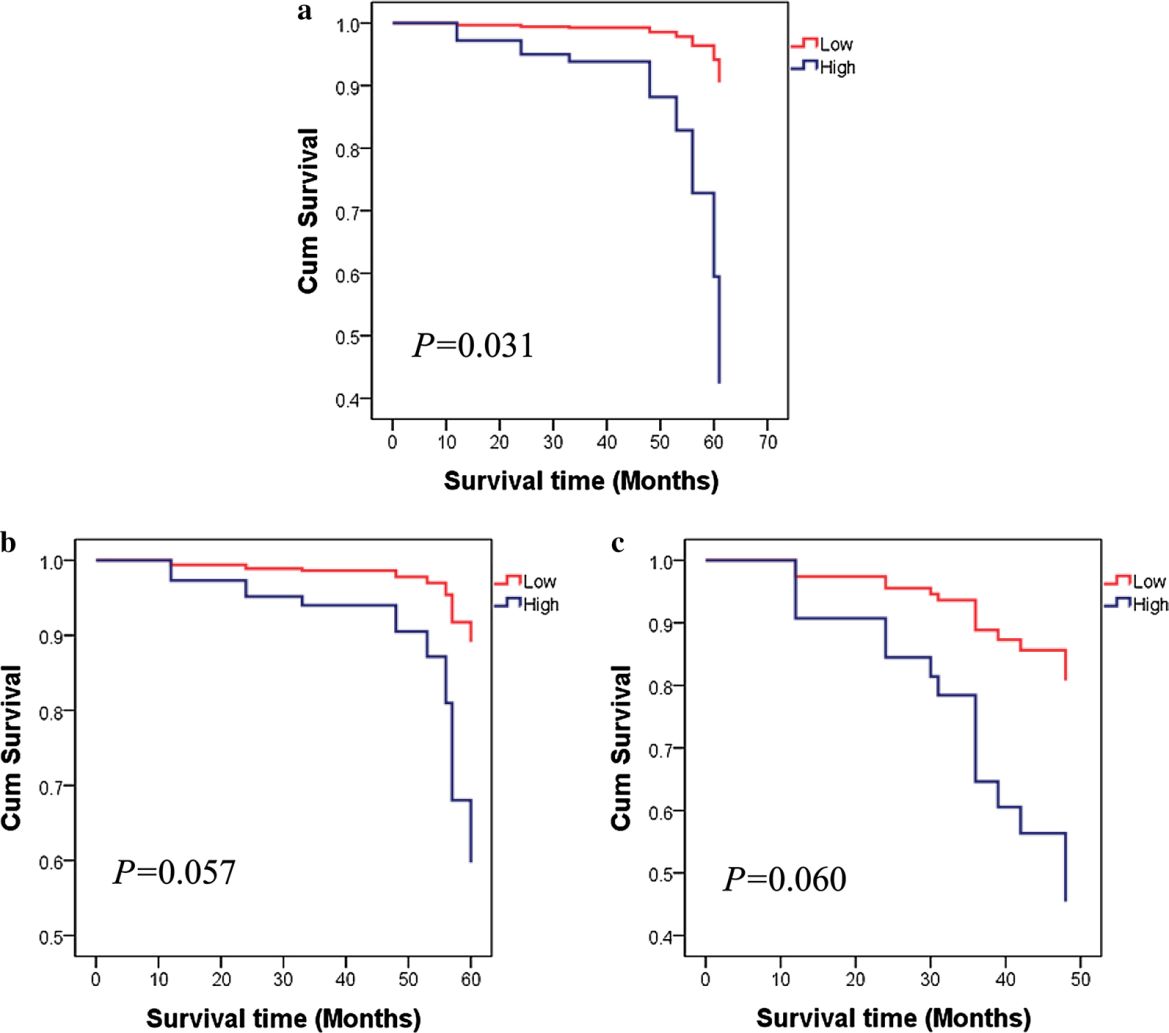
Low expression of XPF was correlated with the prognosis in CRC patients. **a** patients in T1-2 invasive extent with low XPF expression exhibited longer survival time than those with high XPF expression; **b** TNM stage II individuals who expressed lower XPF protein demonstrated better prognosis; **c** patients with vessel carcinoma embolus also identified XPF expression as a bad indicator for CRC prognosis

In this study, the immunohistochemical score 1.8 was used as the cutoff value. To explore whether XPF was a prognostic indicator for patients with CRC, the association of XPF protein expression and CRC overall survival was assessed and summarized in Table 5. As shown in Fig. 6, stratified analysis showed that in individuals whose invasive extent were T1-2, low XPF expression was significantly correlated with better survival rate (95% CI 1.208-52.673, HR = 7.978, P = 0.031). Otherwise, there’s a trend that patients who were in II TNM stage with low XPF expression had better survival (P = 0.057), though the statistical difference was not significant. Patients with vessel carcinoma embolus had the same tendency (P = 0.06).

**Table 5:** Correlation between XPF expression and survival in CRC

	Case	Cases of events	MST	Univariate			Multivariate		
				HR	95% CI	P	HR	95% CI	P
XPF expression									
Low	167	54	50						
High	81	20	48	0.884	0.528–1.478	0.638	1.227	0.668–2.255	0.509
Stratification									
Sex									
Male									
Low	90	28	49						
High	53	15	48	0.968	0.516–1.815	0.919	1.288	0.603–2.752	0.513
Female									
Low	77	26	52						
High	28	5	48	0.767	0.291–2.025	0.592	1.035	0.294–3.635	0.958
Age									
≤ 60									
Low	86	36	49						
High	33	7	47	0.726	0.320–1.647	0.443	0.796	0.233–2.723	0.716
> 60									
Low	81	18	51						
High	48	13	51.5	1.263	0.619–2.579	0.521	1.541	0.723–3.285	0.263
Smoking									
Yes									
Low	37	14	49						
High	22	7	41.5	1.016	0.407–2.535	0.972	0.605	0.109–3.346	0.564
No									
Low	130	40	50						
High	59	13	49	0.835	0.445–1.564	0.573	1.264	0.596–2.682	0.541
Drinking									
Yes									
Low	25	10	49						
High	15	2	49	0.333	0.073–1.519	0.155	0	0.000–(3.315E + 126)	0.899
No									
Low	142	44	50						
High	66	18	48	1.109	0.637–1.931	0.714	1.46	0.750–2.843	0.265
Location									
Colon									
Low	61	19	51						
High	21	9	51	1.485	0.671–3.286	0.329	2.373	0.849–6.636	0.099
Rectum									
Low	106	35	49						
High	60	11	48	0.652	0.330–1.287	0.218	0.748	0.293–1.911	0.544
TNM stage^a^									
I									
Low	3	1	61						
High	3	1	48	65.289	0.000–628084630.4	0.61	13.593	0.000–(4.626E + 22)	0.918
II									
Low	55	21	48						
High	34	8	46.5	0.794	0.349–1.808	0.583	4.1	0.956–17.581	0.057
III									
Low	96	28	52						
High	36	8	51	0.789	0.359–1.731	0.554	0.952	0.411–2.208	0.91
IV									
Low	11	4	39						
High	7	3	42	1.226	0.273–5.509	0.791	3346.862	0.000–(3.231E + 188)	0.97
Invasive extent^a^									
T1-2									
Low	28	7	48.5						
High	16	6	47.5	2.483	0.750–8.222	0.137	7.978	1.208–52.673	*0.031*
T3-4									
Low	107	31	51						
High	48	11	51	0.799	0.401–1.592	0.524	0.958	0.466–1.973	0.908
Lymph node metastasis									
Yes									
Low	108	34	49						
High	40	11	47	1.002	0.507–1.980	0.995	1.462	0.694–3.081	0.318
No									
Low	59	20	54						
High	41	9	51	0.842	0.381–1.861	0.672	0.439	0.096–2.009	0.289
Distant metastasis									
Yes									
Low	39	12	49						
High	25	7	47	1.253	0.487–3.222	0.64	2.592	0.650–10.341	0.177
No									
Low	128	42	50						
High	56	13	48.5	0.773	0.415–1.441	0.418	1.112	0.531–2.332	0.778
Perineural invasion									
Yes									
Low	96	27	49						
High	52	12	48	0.851	0.431–1.681	0.643	1.362	0.608–3.052	0.452
No									
Low	71	27	55						
High	29	8	49	0.907	0.411–2.002	0.81	1.068	0.355–3.209	0.907
Vessel carcinoma embolus									
Yes									
Low	43	9	48						
High	17	7	42	2.241	0.826–6.083	0.113	3.805	0.944–15.333	0.06
No									
Low	124	45	52						
High	64	13	50	0.666	0.359–1.237	0.198	0.82	0.374–1.794	0.619
Growth patten									
Infiltrative									
Low	111	33	49						
High	41	10	48	0.985	0.484–2.005	0.966	1.18	0.523–2.662	0.69
Nested/cloddy									
Low	56	21	52.5						
High	40	10	48	0.767	0.361–1.631	0.49	0.824	0.290–2.340	0.716
Differentiation degree									
Low									
Low	60	22	49						
High	20	4	48.8	0.539	0.183–1.586	0.262	0.939	0.240–3.673	0.928
High									
Low	107	32	50						
High	61	16	48	1.029	0.562–1.882	0.927	1.531	0.701–3.343	0.286
Family history									
Yes									
Low	26	10	54						
High	17	5	47	1.249	0.409–3.820	0.696	8.39E + 15	0.000–(3.821E + 081)	0.635
No									
Low	141	44	49						
High	64	15	49	0.829	0.461–1.491	0.531	1.218	0.622–2.384	0.565

*CI* confidence interval, *HR* hazard radio, *MST* median survival time, *IS* the immunohistochemistry score

The italics values: P < 0.05

^a^Incomplete information

### Functional enrichment analysis of XPF and its interacting genes

**Fig. 7:**
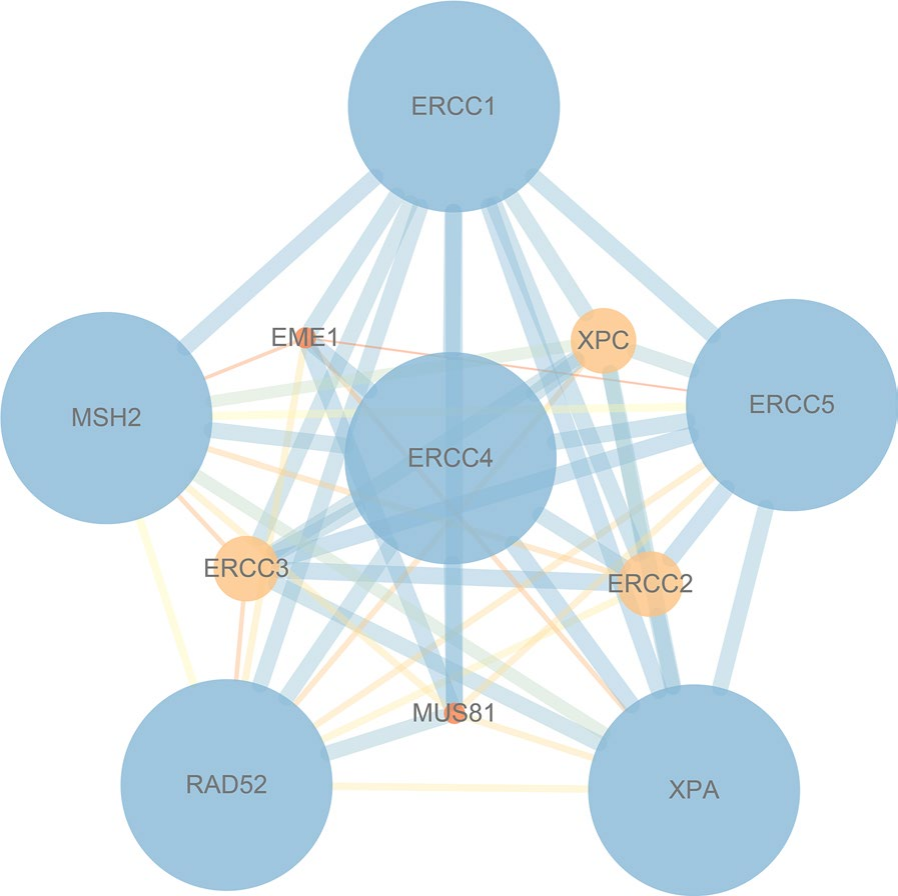
PPI network of XPF. The listed proteins are shown in the results: ERCC1, MSH2, RAD52, XPA, ERCC5, ERCC4, EME1, ERCC3, MUS81, ERCC2, XPC

**Fig. 8:**
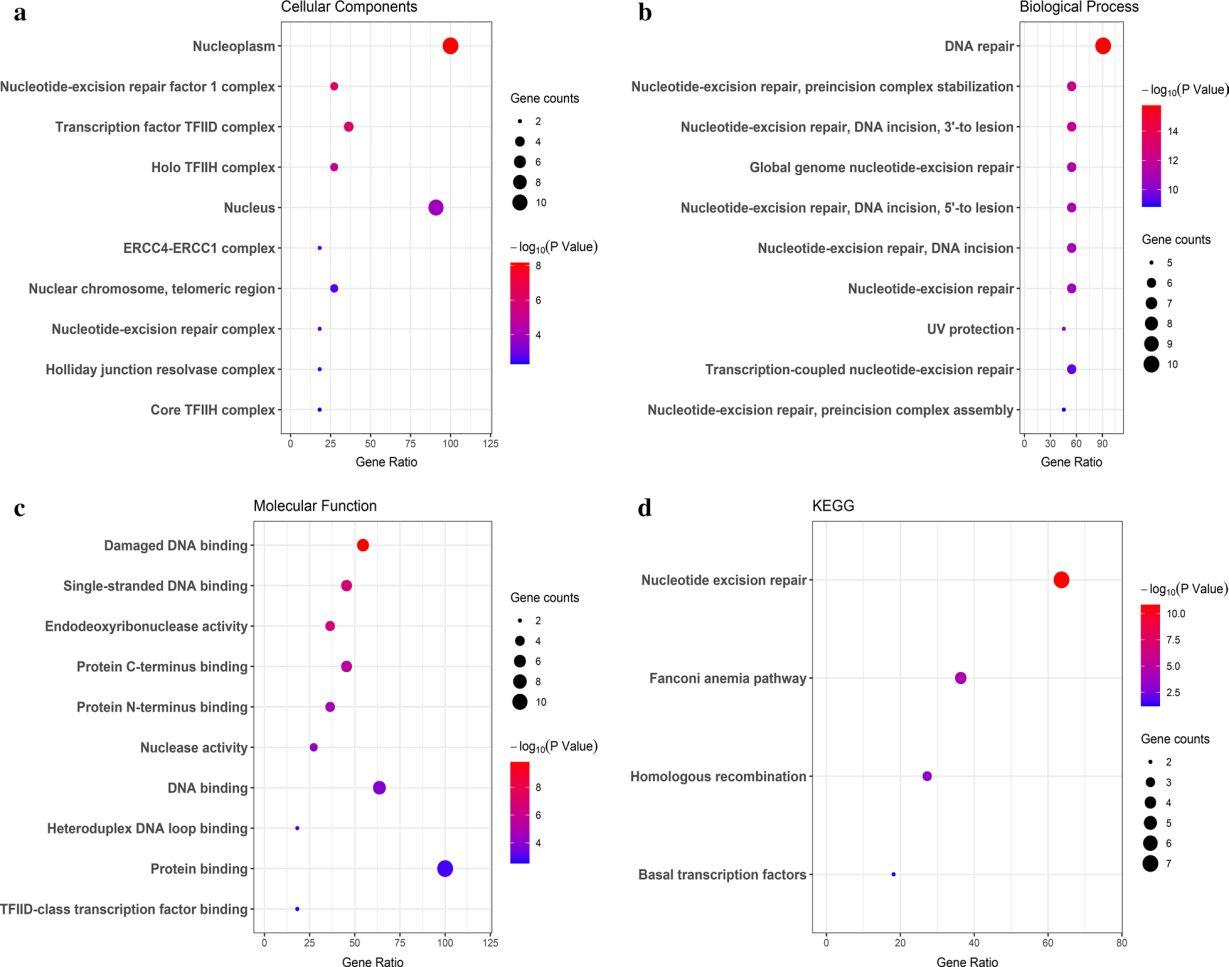
GO and KEGG enrichment of XPF and its interacting genes. **a** Cellular components results of GO analysis; **b** Biological process results of GO analysis; **c** Molecular function results of GO analysis; **d** KEGG analysis results

**Fig. 9:**
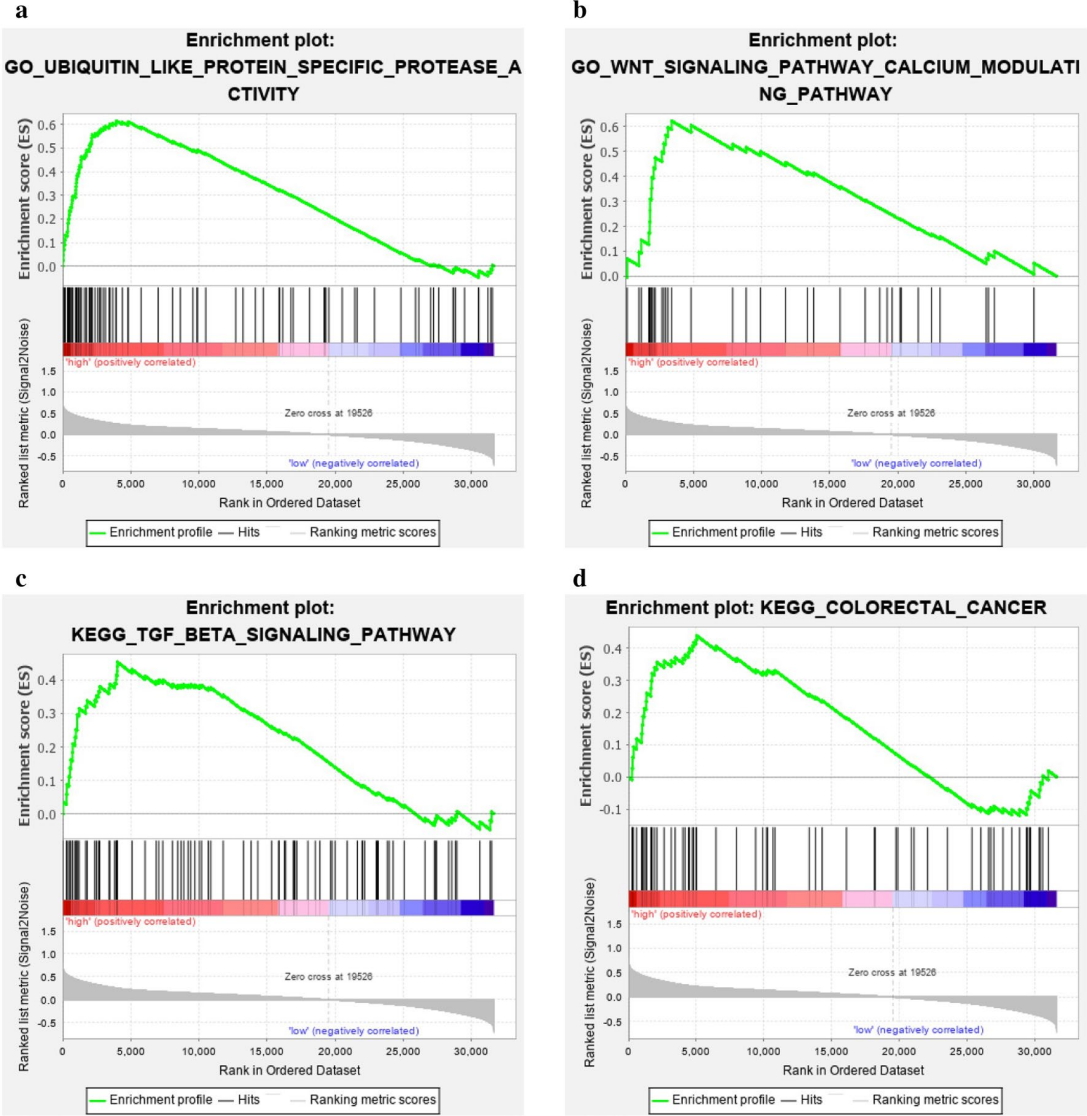
Enrichment analysis results of GSEA. **a** GO-Ubiquitin like protein specific protease activity; **b** GO-Wnt signaling pathway calcium modulating pathway; **c** KEGG-TGF-BETA signaling pathway; **d** KEGG-Colorectal cancer

We analyzed XPF and its interacting genes in STRING (Fig. 7) database. As shown in Fig. 8a–c, GO analysis showed that XPF and its interacting genes were mainly correlated with nucleoplasm, nucleotide-excision repair factor 1 complex, DNA repair, nucleotide-excision repair, preincision complex stabilization, damaged DNA binding single-stranded DNA binding and so on. As for KEGG analysis, these genes were mainly enriched in nucleotide excision repair pathway (Fig. 8d). The results of GSEA for GO analysis (Fig. 9a, b) showed that XPF expression was associated with Ubiquitin like protein specific protease activity, microtubule organizing center organization, cytoplasmic stress granule, peptide-n-acetytransferase activity, centriole, ciliary basal body, microtubule organizing center localization, WNT signaling pathway, calcium modulating pathway and so on. The results of GSEA for KEGG analysis showed that XPF expression was associated with Ubiquitin mediated proteolysis, oocyte meiosis, oocyte meiosis, TGF-β signaling pathway, renal cell carcinoma, adherens junction, long term potentiation, progesterone mediated oocyte maturation, small cell lung cancer, colorectal cancer, WNT signaling pathway and so on (Fig. 9c, d). Heat map of GO and KEGG results of GSEA was showed in Additional file 2: Figure S1.

## Discussion

As a key gene of NER system, XPF plays an indispensable role in keeping the integrity and stability of genome, thus influencing the occurrence of cancer [13]. Although there have been some studies on the correlation between XPF expression and CRC [10–13, 23], this is the first research on XPF expression that covers dynamic CRC development. In addition, this study explored the relationship between XPF expression and clinical traits of CRC. Our results showed that XPF expression was upregulated in CRC tissue compared with adjacent non-tumor tissue, adenoma and anal benign disease. Overexpression of XPF was related to poor prognosis of CRC patients with T1-2 invasive extent. XPF expression was associated with Ubiquitin like protein specific protease activity WNT signaling pathway and so on.

Firstly, we detected the expression of XPF in cancerous and non-cancerous tissue in multiple dimensions and different levels. It is found that the XPF protein expression was significantly higher in CRC tissue than that in adjacent colorectal tissue. The mRNA level of XPF expression came out with similar results: XPF was highly expressed in colonadenocarcinoma than in colon and rectal normal tissue. Subgroup analysis revealed significant difference in male, female, age ≤ 60, age > 60, smoking, no smoking, colon cancer, rectum cancer, drinking, no drinking, lymph node metastasis and other clinicopathological factors. The consistent results of stratified analysis showed that the expression of XPF in CRC was higher than that in adjacent non-tumor tissue regardless of other factors. Previous studies of XPF expression in other tumors have yielded similar results: Li.P et al. [7] found a significant increase of the XPF expression in GC tissue compared with adjacent tissue. Meanwhile, XPF protein played a vital role in the occurrence and progress of GC [7]. Moreover, our results indicated that XPF expression showed an obvious trend of increasing with the development from anal disease, adenoma to CRC. Most sporadic CRC develops from intestinal adenoma. Adenomas represented by conventional, tubular, or tubulovilious adenomas are considered as precancerous lesions of CRC [24]. We conjecture that DNA damage accumulates more along with the dynamic process from normal intestinal tissue to adenoma to CRC. When CRC occurs, cells need the NER system for damage repair, and a large amount of XPF is required to be highly expressed in CRC. As a result, XPF can be a potential biomarker for CRC risk.

Further analysis combined with clinicopathological features of patients brought to light that increased expression of XPF was closely related to clinical features, including rectal cancer and cloddy/nested pattern. XPF expression was related to the invasion of hepatic capsules and microvascular tumor embolus in human hepatocellular carcinoma [25]. The expression of XPF was significantly related to some clinical features such as family history and Laurén classification in GC [7]. XPF abundance was associated with positive ER status in breast cancer, through clinicopathological parameter analysis [26]. Therefore, XPF may have a certain significance for predicting the biologic activities and the progression of CRC. Our study results demonstrated that XPF expression correlates tightly with growth patterns or positions in cancer. As we know the growth patterns of carcinoma may have an influence on the proliferation and invasion of tumor cells. Besides, we suppose that different growth patterns or tumor site may cause distinct degrees of DNA damage, thereby inducing the translation of XPF with diverse activities, resulting in the differences in XPF expression. Further study is warranted to investigate the relationship between XPF expression and clinopathological features in CRC.

In this study, we also explored the association between XPF expression and prognosis of CRC patients. The results showed that the XPF expression was not significantly associated with the survival time in overall analysis. As for patients in stage T1-T2, those with low XPF expression can survive longer than those with high expression. Previously, low XPF expression was also found to predict better prognosis in other types of cancer. Li et al. reported that XPF-positive patients had shorter survival time than XPF-negative patients in GC [7]. Mesquita et al. found that low ERCC1/XPF expression was related to better progression-free survival in 331 ovarian cancer patients [27]. Vaezi et al. revealed that Low XPF expression correlated with longer survival time in patients of squamous cell carcinoma of the head and neck [8]. It has been reported that XPF-ERCC1 endonuclease is required in the repair pathways such as NER, DSB, ICL responsible for the repair of helix-distorting DNA lesions as well as interstrand crosslinks aroused by radiation and platinum compounds [28, 29]. We may extrapolate that low XPF expression are more sensitive to DNA damage agents such as cisplatin. With the increase of damage in cancer tissue, damage repair activities increase, so the high expression of XPF in colorectal cancer tissue also indicates a worse prognosis. The correlation between low expression of XPF and longer survival time may be applicable to CRC, but its molecular mechanism still needs further research to be clarified.

The expression pattern of XPF in CRC and its potential prognostic role inspired our understanding of XPF in development and progression of CRC. Therefore, we further performed PPI and functional enrichment analysis to reveal the interacting network and the biological function of XPF. By STRING database, we queried the genes interacted with XPF, which were ERCC1, XPA, ERCC5, MSH2, XPC, ERCC3, ERCC2 and so on. Firstly, functional analysis of this PPI network showed that positive regulation of DNA secondary structure binding and damaged DNA binding were the most significant. Besides, the network was also correlated with UV protection and DNA repair complex. XPF is an essential human gene in the NER pathway responsible for the removal of UV-C photoproducts and large volume adducts from DNA [4]. KEGG analysis also showed that XPF-related PPI network was mainly enriched in nucleotide excision repair pathway. Studies have mentioned that XPF was in charge of the 5′ incision process in the NER pathway [27]. GSEA analysis showed that with the expression of XPF increased, some pathways can be activated, such as ubiquitin like protein specific protease activity, WNT signaling pathway and calcium modulating pathway. There are reports in the literature showing that high expression of ubiquitin-specific protease 6 N-terminal-like protein can regulate proliferation activity of CRC cell via Wnt/β-catenin pathway [22]. Therefore, we suspected that increased XPF expression may be related to CRC risk and progression by activating the above mentioned pathways.

## Conclusions

In conclusion, we investigated the expression of XPF in adjacent non-tumor tissue, benign disease, adenoma and CRC by immunohistochemistry. We found that the expression of XPF was gradually increased with the progress of CRC. Besides, XPF protein expression was associated with tumor location and growth patterns of CRC. XPF may be a promising biomarker for CRC risk, and also showed potential as a prognosis predictor in T1-T2 stage patients with CRC.

## Supplementary information

**Additional file 1: Table S1.** XPF expression in CRC and anal disease tissue. Mann–Whitney U-test of nonparametric test to compare the XPF protein expression between CRC and adjacent tissue. PR, positive rate. Negative (−), light positive (+), positive (++), strong positive (+++) staining. The bold values: P < 0.05.

**Additional file 2: Figure S1**. Heat map of GO and KEGG results of GSEA: **a** GO-Ubiquitin like protein specific protease activity; **b** GO-Wnt signaling pathway calcium modulating pathway; **c** KEGG-TGF-BETA signaling pathway; **d** KEGG-Colorectal cancer.

## Data Availability

The authors declare that the data supporting the conclusion of this study are included within the article.

## Abbreviations

CRC: Colorectal cancer
NER: Nucleotide excision repair
GC: gastric cancer
UICC: International Union Against Cancer
AJCC: United Joint Cancer Committee
HNPCC: Hereditary nonpolyposis colorectal cancer
GO: Gene ontology
KEGG: Kyoto Encyclopedia of Genes and Genomes
GSEA: Gene Set Enrichment Analysis
MST: Median survival time
IS: Immunoreactivity score

## References

[1] Iyama T, Wilson DM (2013). DNA repair mechanisms in dividing and non-dividing cells. DNA Repair.

[2] Slyskova J, Korenkova V, Collins AR, Prochazka P, Vodickova L, Svec J, Lipska L, Levy M, Schneiderova M, Liska V (2012). Functional, genetic, and epigenetic aspects of base and nucleotide excision repair in colorectal carcinomas. Clin Cancer Res.

[3] de Laat WL, Jaspers NG, Hoeijmakers JH (1999). Molecular mechanism of nucleotide excision repair. Genes Dev.

[4] Liu J, He C, Xing C, Yuan Y (2014). Nucleotide excision repair related gene polymorphisms and genetic susceptibility, chemotherapeutic sensitivity and prognosis of gastric cancer. Mutat Res.

[5] Enzlin JH, Scharer OD (2002). The active site of the DNA repair endonuclease XPF-ERCC1 forms a highly conserved nuclease motif. EMBO J.

[6] Zhang Q, Shi J, Yuan F, Wang H, Fu W, Pan J, Huang Y, Yu J, Yang J, Chen Z (2016). Higher expression of XPF is a critical factor in intrinsic chemotherapy resistance of human renal cell carcinoma. Int J Cancer.

[7] Li P, Ma Y (2018). Correlation of xeroderma pigmentosum complementation group F expression with gastric cancer and prognosis. Oncol Lett.

[8] Vaezi A, Wang X, Buch S, Gooding W, Wang L, Seethala RR, Weaver DT, D’Andrea AD, Argiris A, Romkes M (2011). XPF expression correlates with clinical outcome in squamous cell carcinoma of the head and neck. Clin Cancer Res.

[9] Chen W, Zheng R, Baade PD, Zhang S, Zeng H, Bray F, Jemal A, Yu XQ, He J (2016). Cancer statistics in China, 2015. CA Cancer J Clin.

[10] Yang H, Li G, Li WF (2015). Association between ERCC1 and XPF polymorphisms and risk of colorectal cancer. Genet Mol Res.

[11] Kabzinski J, Majsterek I, Dziki A, Mik M (2015). The Role of the XPF Gene Polymorphism (Xrcc4) Ser835ser in the Risk of Malignant Transformation of Cells in the Colorectal Cancer. Pol Przegl Chir.

[12] He XF, Liu LR, Wei W, Liu Y, Su J, Wang SL, Shen XL, Yang XB (2014). Association between the XPG Asp1104His and XPF Arg415Gln polymorphisms and risk of cancer: a meta-analysis. PLoS ONE.

[13] Zhang Y, Wu S, Zhou X, Huang F, Chen R, Wang Y, Wu J (2019). Association between nucleotide excision repair gene polymorphism and colorectal cancer risk. J Clin Lab Anal.

[14] Deng N, Liu JW, Sun LP, Xu Q, Duan ZP, Dong NN, Yuan Y (2014). Expression of XPG protein in the development, progression and prognosis of gastric cancer. PLoS ONE.

[15] Rhodes DR, Yu J, Shanker K, Deshpande N, Varambally R, Ghosh D, Barrette T, Pandey A, Chinnaiyan AM (2004). ONCOMINE: a cancer microarray database and integrated data-mining platform. Neoplasia.

[16] Rhodes DR, Kalyana-Sundaram S, Mahavisno V, Varambally R, Yu J, Briggs BB, Barrette TR, Anstet MJ, Kincead-Beal C, Kulkarni P (2007). Oncomine 3.0: genes, pathways, and networks in a collection of 18,000 cancer gene expression profiles. Neoplasia.

[17] Szklarczyk D, Gable AL, Lyon D, Junge A, Wyder S, Huerta-Cepas J, Simonovic M, Doncheva NT, Morris JH, Bork P (2019). STRING v11: protein-protein association networks with increased coverage, supporting functional discovery in genome-wide experimental datasets. Nucleic Acids Res.

[18] The Gene Ontology C: Expansion of the Gene Ontology knowledgebase and resources. *Nucleic Acids Res* 2017, 45(D1):D331-D338. 10.1093/nar/gkw1108 PMC5210579 27899567

[19] Kanehisa M, Furumichi M, Tanabe M, Sato Y, Morishima K (2017). KEGG: new perspectives on genomes, pathways, diseases and drugs. Nucleic Acids Res.

[20] da Huang W, Sherman BT, Lempicki RA (2009). Systematic and integrative analysis of large gene lists using DAVID bioinformatics resources. Nat Protoc.

[21] The Gene Ontology (GO) project in 2006. Nucleic acids research 2006, 34(Database issue):D322-326. 10.1093/nar/gkj021 PMC1347384 16381878

[22] Sun K, He SB, Yao YZ, Qu JG, Xie R, Ma YQ, Zong MH, Chen JX (2019). Tre2 (USP6NL) promotes colorectal cancer cell proliferation via Wnt/beta-catenin pathway. Cancer Cell Int.

[23] Hou R, Liu Y, Feng Y, Sun L, Shu Z, Zhao J, Yang S (2014). Association of single nucleotide polymorphisms of ERCC1 and XPF with colorectal cancer risk and interaction with tobacco use. Gene.

[24] Bae JM, Kim JH, Kang GH (2016). Molecular subtypes of colorectal cancer and their clinicopathologic features, with an emphasis on the serrated neoplasia pathway. Arch Pathol Lab Med.

[25] Liao X, Li Y, Li H, Huang W, Wang H, Xie W (2020). Expression and clinical significance of ERCC1 and XPF in human hepatocellular carcinoma. Onco Targets Ther.

[26] Fagerholm R, Sprott K, Heikkinen T, Bartkova J, Heikkila P, Aittomaki K, Bartek J, Weaver D, Blomqvist C, Nevanlinna H (2013). Overabundant FANCD2, alone and combined with NQO1, is a sensitive marker of adverse prognosis in breast cancer. Ann Oncol.

[27] Mesquita KA, Alabdullah M, Griffin M, Toss MS, Fatah T, Alblihy A, Moseley P, Chan SYT, Rakha EA, Madhusudan S (2019). ERCC1-XPF deficiency is a predictor of olaparib induced synthetic lethality and platinum sensitivity in epithelial ovarian cancers. Gynecol Oncol.

[28] Niedernhofer LJ, Garinis GA, Raams A, Lalai AS, Robinson AR, Appeldoorn E, Odijk H, Oostendorp R, Ahmad A, van Leeuwen W (2006). A new progeroid syndrome reveals that genotoxic stress suppresses the somatotroph axis. Nature.

[29] Ahmad A, Robinson AR, Duensing A, van Drunen E, Beverloo HB, Weisberg DB, Hasty P, Hoeijmakers JH, Niedernhofer LJ (2008). ERCC1-XPF endonuclease facilitates DNA double-strand break repair. Mol Cell Biol.

